# Calcium Identification and Scoring Based on Echocardiography. An Exploratory Study on Aortic Valve Stenosis

**DOI:** 10.3390/jpm11070598

**Published:** 2021-06-24

**Authors:** Luis B. Elvas, Ana G. Almeida, Luís Rosario, Miguel Sales Dias, João C. Ferreira

**Affiliations:** 1 Inov Inesc Inovação—Instituto de Novas Tecnologias, 1000-029 Lisbon, Portugal; Luis_Manuel_Elvas@iscte-iul.pt; 2 Instituto Universitário de Lisboa (ISCTE-IUL), ISTAR, 1649-026 Lisboa, Portugal; miguel.dias@iscte-iul.pt; 3 Faculty of Medicine, Lisbon University, Hospital Santa Maria/CHULN, CCUL, 1649-028 Lisbon, Portugal; amalmeida@medicina.ulisboa.pt (A.G.A.); lsrosario@medicina.ulisboa.pt (L.R.)

**Keywords:** ultrasound images, coronary artery disease, echocardiograms, CT-scan, computed tomography, coronary artery calcium, feature extraction, image classification, computer vision

## Abstract

Currently, an echocardiography expert is needed to identify calcium in the aortic valve, and a cardiac CT-Scan image is needed for calcium quantification. When performing a CT-scan, the patient is subject to radiation, and therefore the number of CT-scans that can be performed should be limited, restricting the patient’s monitoring. Computer Vision (CV) has opened new opportunities for improved efficiency when extracting knowledge from an image. Applying CV techniques on echocardiography imaging may reduce the medical workload for identifying the calcium and quantifying it, helping doctors to maintain a better tracking of their patients. In our approach, a simple technique to identify and extract the calcium pixel count from echocardiography imaging, was developed by using CV. Based on anonymized real patient echocardiographic images, this approach enables semi-automatic calcium identification. As the brightness of echocardiography images (with the highest intensity corresponding to calcium) vary depending on the acquisition settings, echocardiographic adaptive image binarization has been performed. Given that blood maintains the same intensity on echocardiographic images—being always the darker region—blood areas in the image were used to create an adaptive threshold for binarization. After binarization, the region of interest (ROI) with calcium, was interactively selected by an echocardiography expert and extracted, allowing us to compute a calcium pixel count, corresponding to the spatial amount of calcium. The results obtained from these experiments are encouraging. With this technique, from echocardiographic images collected for the same patient with different acquisition settings and different brightness, obtaining a calcium pixel count, where pixel values show an absolute pixel value margin of error of 3 (on a scale from 0 to 255), achieving a Pearson Correlation of 0.92 indicating a strong correlation with the human expert assessment of calcium area for the same images.

## 1. Introduction

The morphology and function of cardiac valves can be assessed in vivo in patients using echocardiography, which is widely used, does not use radiation, and can therefore be repeated throughout one’s life; it has a high temporal and spatial resolution that can evaluate valve morphology and mobility for every cardiac cycle in either 2D tomographic or 3D models; and it can evaluate the valve morphology and mobility for every cardiac cycle, as well in either 2D tomographic or 3D models.

Aortic valve stenosis is the most common cardiac valvular disease and is highly prevalent nowadays [1], affecting 7% of the population over 65 years old. It has a 60% annual mortality rate in untreated severe cases, with survival <5 years when symptoms evolve. The incidence and prevalence of the degenerative type is increasing as this segment of the population grows older [2]. It is estimated that 2,262,325 people are at risk in Portugal, accounting for 22% of the population [3]. According to the European Society of Cardiology’s recommendations for diagnosing and treating aortic stenosis, echocardiography is the first-line method to make the diagnosis and monitor the patient and valve calcification is a main feature to assess severity. The current European guidelines for diagnosing and treating aortic stenosis recommend also echocardiography as a first-line method to establish every patient’s diagnosis, and repeat echocardiography every 6 months for severe cases or yearly for the moderate disease [4]. Consequently, a standardized diagnostic tool is needed to diagnose, assess the severity of the stenosis, and follow-up this large population.

Computed tomography (CT) provides a calcium quantification method, expressed as a calcium score, based in the Agatston method [5]. When applied to aortic valve stenosis, this calculation is useful since calcification is a hallmark of this disease, and it has been shown that the amount of valve calcification is related to disease severity [6]. The severity of stenosis is directly related to prognosis and has an impact on the decision to replace the valve. This is particularly important when assessing severity with echocardiography, which may be difficult or debatable in up to 20% of cases [7], namely in cases of a low-flow low-gradient when the amount of calcium is often calculated in order to establish the stenosis severity. Previous studies have shown the value of cardiac CT-scans for determining the aortic calcium score, which is the only current imaging modality available for this purpose. Nevertheless, this approach bears costs, not only monetary, but from health too, since it is an ionizing technique which uses radiation to extract the amount of calcium [8], which may have long-term effects on the health condition of the patient. The development of a fully automated method able to identify and quantify the amount of valve calcium using echocardiography should be most valuable due to the non-ionizing character of this technique as well as the possibility of repeatability and long-term follow-up.

Before performing a CT-scan to obtain a calcium score, the calcium is first identified from the early stages of the disease by echocardiography [4], a non-invasive non-radiation method that uses ultrasound to scan the heart.

The standard of calcium detection is visual and requires training from medical professionals. The process is dependent on human performance and quantitation based in visual methods is time-consuming and prone to estimation errors. Moreover, when using ultrasound, the results may depend on the settings used for the image acquisition. Previous studies have addressed calcium quantification by echocardiography using commercial software for the identification of calcium. Using CT calcium scores as a reference, correlations between the two modalities was only moderate, with debatable usefulness for clinical application [9].

In fact, there are several approaches to this problem, such as adopting Machine Learning (ML) techniques. An example of this approach in the healthcare field can be seen in predicting the probability of lethal pneumonia to optimize costs, manage low-risk patients as outpatients, and to hospitalize high-risk patients [10]. A key ML technique, Convolutional Neural Networks (CNN), is the engine behind many of the recent advances in the field. A major drawback of CNN-based approaches is that it inherently works as a ‘black box’, with little visibility into the rationale and explanation of the classification decision provided by CNN [11]. As a black-box metaphor, CNN suffers from a lack of human interpretability, which is fundamental in understanding the methods’ operation. Besides, implementing CNNs requires large amounts of labeled data to meet the technique training requirements [12], which is out of the scope of this exploratory study, forcing us to look for alternative methods.

A Standard Computer Vision technique was selected as the best option, since it brings the added advantage of a supporting explanation. With this approach, we propose a binarization of the grey-level echocardiography image input, with an adaptive image threshold technique for image segmentation, where in the end, the binary image results in a white foreground (the calcium regions), with all other anatomic structures in black. This model should be able to identify aortic valve calcification and obtain a quantification of the pixel’s intensity and white pixel count, proportional to the amount of calcium, in parallel to a CT-scan calcium scan analysis.

This study aims to develop and evaluate a Computer Vision model/algorithm applicable to echocardiographic images, via adaptive image segmentation of echocardiography imaging, avoiding a Machine Learning approach and access to large amounts of labeled data, for identifying and quantifying the amount of calcium in aortic valve stenosis, in a fully automated process.

## 2. Materials and Methods

Our artifact envisages the identification of the presence of calcium in the aortic valve. Several image enhancement processes were tested, aiming to highlight the areas with a high concentration of calcium, which are described in the next sections. Our main goal is to develop an approach that can be implemented in hospital to perform a semi-automatic approach of calcium score from echography to reduce work from doctors and reduce the need for CT-scans.

### 2.1. Methodology

A systematic literature review was made by following the PRISMA (Preferred Reporting Items for Systematic Reviews and Meta-Analysis) Methodology [13], and with the following research question: “What is the state of the art of analyzing Ultrasound and CT-scan imaging, to find the calcium score of the aortic valve?”.

Information Systems (IS) research risks losing leverage over the fields where its applicability is critical if it lacks a strong component that provides applied research solutions [14]. The IS study is characterized by two major paradigms.

On one side, there’s behavioral science, which tries to come up with theories that predict personal or organizational conduct. On the other hand, design-science seeks to extend human and organizational capacities by developing creative artifacts [15].

In light of this, the Design Science Research Methodology (DSRM) and the six principles suggested by Peffers et al. [14] are used in the development of this article. This approach has its roots in engineering and artificial sciences, and its main goal is to create relevant artifacts that add value to the fields in which they are used. 

The DSRM process has four different entry points, also known as methods, the first one being used in this research work, Problem-Centered-Initiation, since it is, by definition, the starting point of our methodology.

Since DSRM takes a problem-solving approach, it is critical to evaluate the artifacts to provide feedback and a better understanding of their problems, emphasizing in the improving of both their quality and design in subsequent iterations of the process. Before the final artifact is produced, this build-and-assess loop is normally repeated several times [16].

The first two activities are introduced in the Section 1, the third activity—Design and Development—is explained and demonstrated in Section 2, as well as the evaluation of the first results representing the robustness of our artifact. The evaluation is made in Section 3.6. The last activity—communication—is achieved in this article.

### 2.2. Echocardiography Binarization Process

Our developments adopted the OpenCV library [17]. In the first stage, the image histogram was equalized in order to improve the contrast of the image and stretch the intensity range, using the “equalizeHist” function [18]. This equalization relies on the mapping of one distribution to another distribution—a more uniform and wider distribution of the pixel intensity values—to spread the intensity values over the whole range. For the histogram of the input image $H_{\left(i\right)}$, its cumulative distribution ${H^{\prime }}_{\left(i\right)}$ is:

(1) $${H^{\prime }}_{\left(i\right)}=\sum_{0\leq j<i}H_{\left(j\right)},$$

where *i* is the intensity values from the given histogram and j the more uniform distribution of intensity values.

To use this as a remapping function, ${H^{\prime }}_{\left(i\right)}$ has to be normalized. Since the pixel grayscale intensities go from 0 to 255, the new intensity values of the equalized image can be obtained by applying the following remapping function to the source echocardiography image, $src\left(x,y\right)$:

(2) $$equalized\left(x,y\right)=H^{\prime }\left(src\left(x,y\right)\right),$$

**Figure 1:**
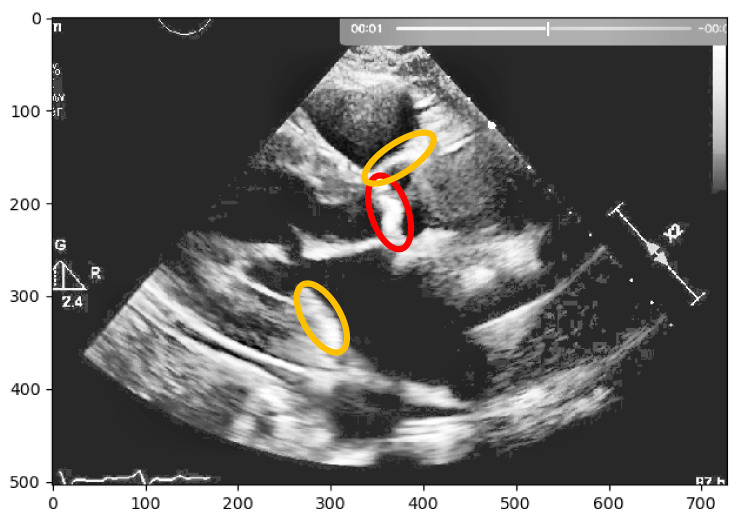
Echocardiography image with CLAHE—The red circle represents the region of interest (ROI) where the calcified aortic valve is located, and in yellow, other structures are marked, which are non-calcified structures.

Subsequently, to improve the contrast of $equalized\left(x,y\right)$, a Contrast Limited Adaptive Histogram Equalization algorithm [19] was implemented that will divide the image into several non-overlapping regions of almost equal sizes, creating several histograms that will redistribute the image brightness, achieving the results in the overall image contrast depicted in Figure 1. To conclude the process, a thresholding technique was used, to segment the image into foreground and background, for further interpretation.

Nevertheless, this simple approach relying solely on histogram equalization leads to poor and inconclusive results in terms of visualizing and extracting the presence of calcium, as shown in Figure 1. The red circle represents where there is calcium on the aortic valve, and in yellow, other structures are marked, which are indistinguishable from each other.

**Figure 2:**
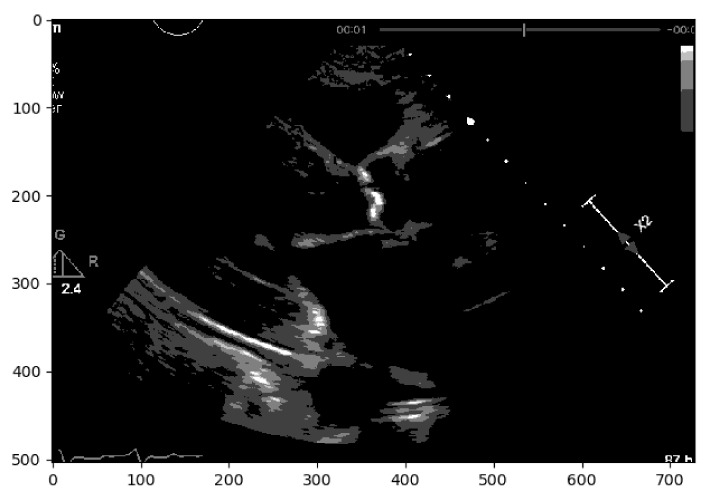
Echocardiography image with region-based segmentation.

**Figure 3:**
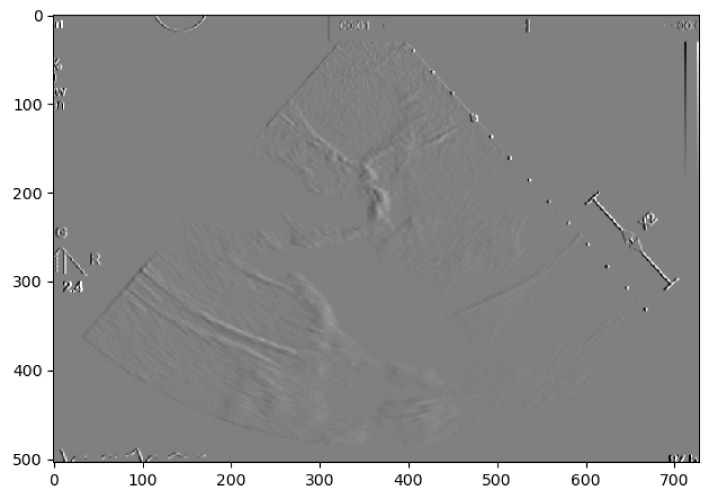
Echocardiography image with edge detection.

In a second approach, a Region-based Segmentation [20] was attempted, where the aim was to segment different objects (calcium/non-calcium) by analyzing their pixel values. This technique classifies the pixels—based on a threshold applied to each pixel value—as an object or background. Moreover, since we may have multiple objects—given that calcium can go from severe to none in different scale values—multiple thresholds were initially defined to segment multiple objects, as represented by Figure 2. However, if we have an image with no significant grayscale difference, this approach will fail to get accurate segments. To mitigate this issue, another approach was attempted to have a more comprehensive and interpretable image. An Edge Detection algorithm [21] was used where the pixel brightness is scaled to an embossed image, where the height of each “mountain” corresponds to the pixel brightness. Figure 3 shows us the application of Edge Detection on an echocardiography image of the left ventricle. This approach turned out to be redundant since it represents the pixel values by “mountains heights”. This could be immediately calculated if the first step were extracting the pixel’s exact value and minimizing computation time. Otherwise, after implementing this algorithm, it would be another one would be needed to find each “mountain” height.

After the above-described initial approaches to our problem, it was concluded that instead of focusing our interest on mimicking human eye comprehension of the calcium presence, we could address our challenge in a different way, a third approach, by extracting the region of interest’s pixel values and seeing how they correlate with the amount of calcium present in the aortic valve.

In a first step, image binarization [22] was performed with a fixed threshold of 140 in the pixel grayscale value (in a scale from 0 to 255), where the pixels with an intensity above 140 were transformed in white (255), and the remaining in black (0), thus helping to identify the regions where there is a presence of calcium.

**Figure 4:**
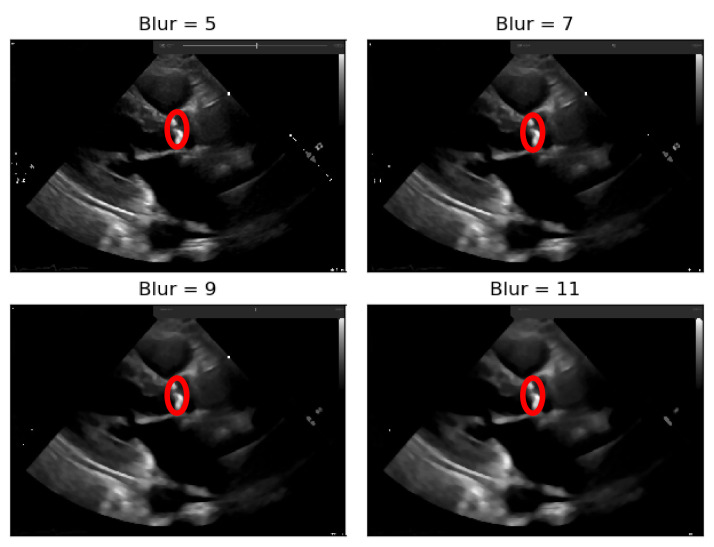
Echocardiography image with four levels of blurring applied—the red circle represents our ROI where the aortic valve is located.

To deal with some natural constraints in terms of noise that characterize echocardiography imaging, particularly the process of sampling still images from the echocardiography video, different blurring treatments were performed to clean some of the image’s noise due to the echocardiography’s motion. Blurring an image will average rapid changes in the different pixel intensities, and this corresponds to a low-pass filter applied to the image [23], which removes noise while leaving the majority of the image structures still present in the image as depicted in Figure 4.

As it can be seen from Figure 4, when the Blur = 11 (experimentally adjusted with trial and error)), it can be easily identified, visually, in the regions where there is a presence of calcium (identified by the red circles). On this operation the central element of the image is replaced by the median of all the pixels in the kernel region, where the 11 means that it takes into consideration a kernel of 11 by 11.

**Figure 5:**
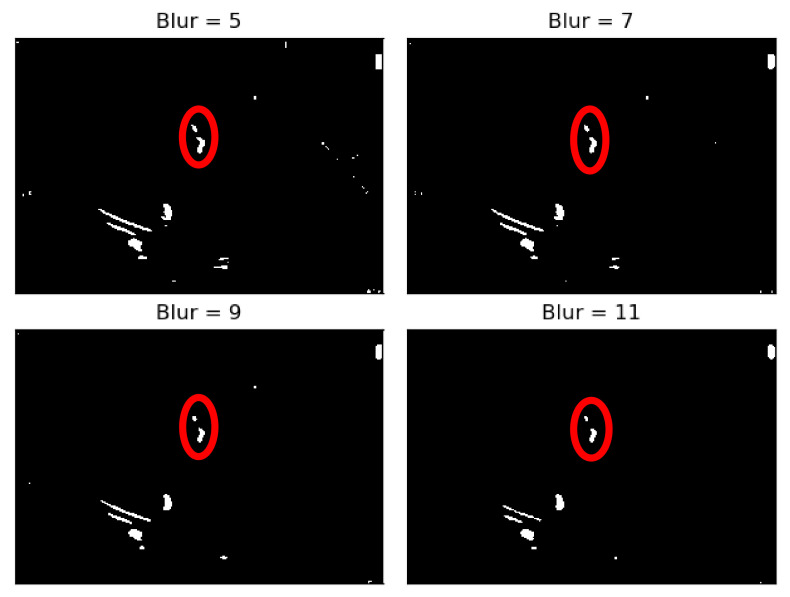
Binarization of an echocardiography image, for each size of the kernel parameter applied—The red circle represents our ROI (aortic valve).

To the resulting images of this blurring phase, a binarization operation with a fixed pixel threshold value of 160 was produced, experimentally obtained by analyzing 48 cases of echocardiography images, where 255 corresponds to calcium, as seen in Figure 5. This initial approach of a using a fixed threshold is not sufficient for our problem at hand, since our images’ brightness may vary, given different data collection conditions. To tackle this issue, an adaptive binarization technique has to be performed, which will be further explained.

In Figure 5, it is noticeable that when the blurring parameter increases from 5 to 11, we get a cleaner image (without small white dots—noise). However, we can also notice that in the region of interest (marked with red circles), when the blur increases, we lose pixels, since the region gets smaller. To mitigate this, a mask dilation operator was applied to each region of interest.

**Figure 6:**
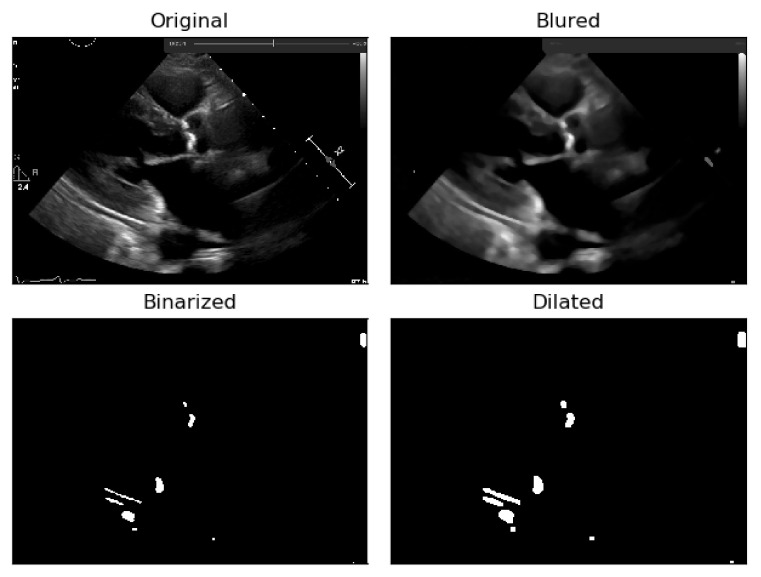
Application of the dilation mask to the regions of interest of the image, in order to recover the pixels lost in the blurring phase.

As shown Figure 6, the pixels lost in the blurring phase can be recovered by applying the dilation mask to the regions of interest in the image.

The next phase was to make our binarization adaptive and not based solely on a fixed threshold (initially set to 160), given the high variability in the imaging data collection procedures.

### 2.3. Adaptive Binarization Process

To achieve this adaptive binarization based on a fixed threshold, we need to confer to it a normalization value, in order to adapt to the various image changes resulting from the settings applied to the echocardiographic image acquisition.

The echocardiographic image suffers two steps of processing: (1) a post-processing stage where gains are imposed in the image after data acquisition, defined by windowing or grey-scale mapping, using the window width (WW) and level (WL) (2) and followed by an image analysis stage performed by the specialist.

#### 2.3.1. Post-Processing Normalization

**Figure 7:**
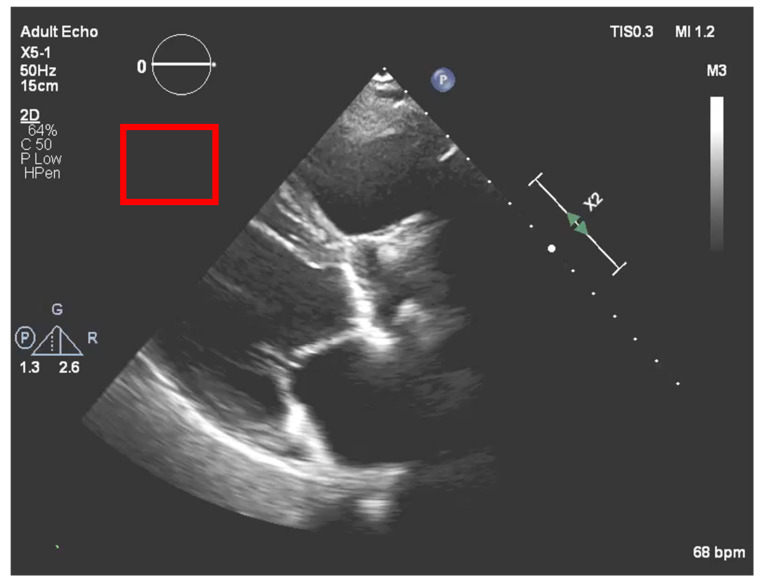
Normalization region of interest (red square) to compensate the post image processing.

After the echocardiography raw data acquisition, gains are added to the image in a post-processing procedure. In our process, it is necessary to compensate for the new brightness that the image acquires by such a process. To accomplish this, a region outside the ultrasound sector was selected that would act as a normalization boundary of “dark” regions, as represented in Figure 7.

Once we had this sub-matrix, we subtracted the mean of its values from our calcium ROI pixel values, thus compensating for the image gain of this stage.

**Figure 8:**
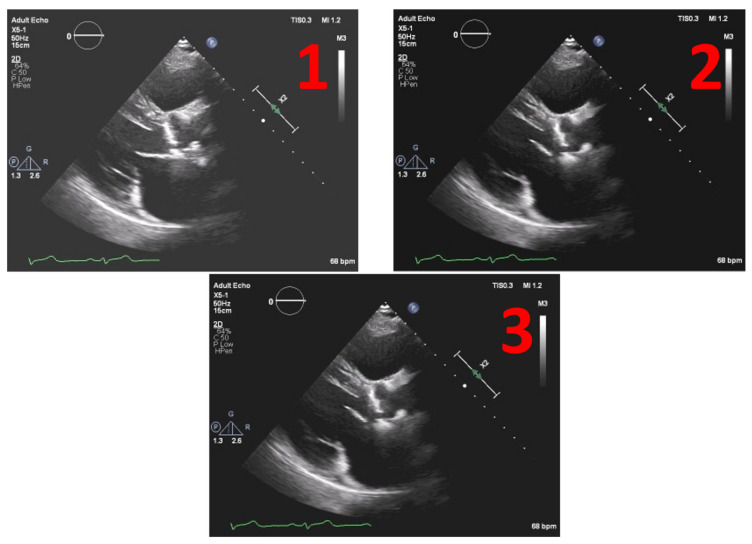
Echocardiography examples with different Windrow Levels (WL) and fixed Window Width of 250 (**1**) WL = 75, (**2**) WL = 100, and finally, (**3**) WL = 125.

This method was tested on several images. Figure 8 depicts the example of one echocardiography with the most used different types of gains set in a post-processing stage, with the settings Window Width fixed at 250 and Window Level (WL) permuting between 75, 100 and 125.

**Figure 9:**
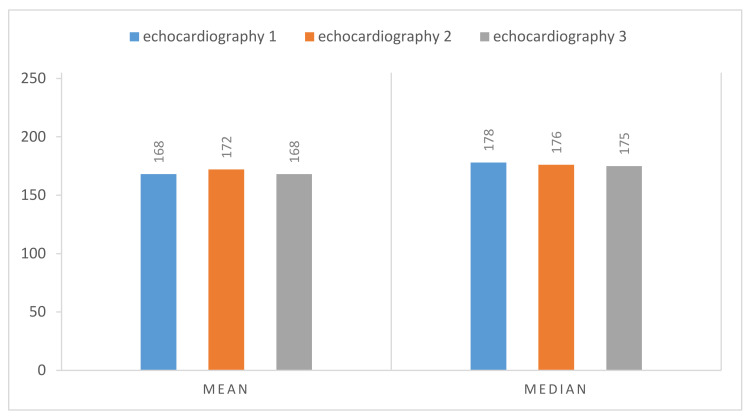
Threshold values of pixel intensity for calcium, extracted from echocardiographic images with different post-processing gains. Windrow Levels (WL): (1) WL = 75, (2) WL = 100, and finally, (3) WL = 125, all with a fixed Window Width of 250.

In Figure 9 we have the normalized result of the calcium threshold obtained with these representative echocardiography cases, showing coherent results, extracted from the calcium present in the aortic valve, where we have the mean and the median of the values extracted from our ROI, the scale being from 0 to 255 (the greyscale pixel values). We can see that the values of pixels intensity extracted have a low absolute variation, suggesting the validity of our model since we have the same image with different gains.

#### 2.3.2. Ultrasound Settings Normalization

After post-processing (prior stage), we proceeded with a second normalization process, related to the ultrasound settings that alter the image brightness and contrast during the echocardiography raw data acquisition, and which have direct consequences on the echocardiography pixel’s intensity levels. To mitigate this, we needed to interactively find a darker region of the echocardiography (inside the ultrasound range) and consider the mean of the values of that region as a “black threshold”, a reference area. By means of a manual step performed by the specialists, an area of the image was interactively selected from a structure which was expected to have very low brightness, typically corresponding to blood flow and subject to the same acquisition and post-processing (window width and level).

**Figure 10:**
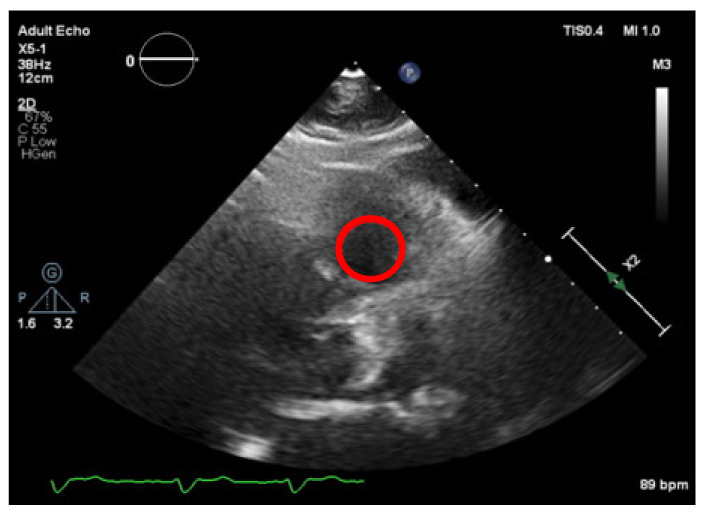
Normalization region (right ventricle cavity).

**Figure 11:**
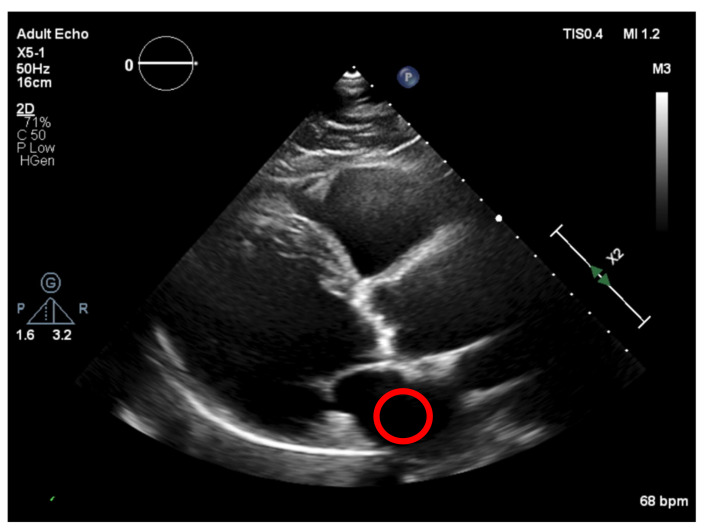
Normalization region (left atrium cavity).

Firstly, the right ventricle cavity was chosen as a potential candidate for a reference ROI, given its minimal signal refractions. An ROI is selected in this image (as shown in Figure 10), corresponding to a darker region and allowing us to define the “dark” level of the image, by taking the mean of the values in that ROI) and then normalizing all pixel values of the image, by subtracting the “dark” value from their values. This would create a dynamic threshold, changing every time the brightness varies due to modifications in the ultrasound properties. With the ROI placed at the left atrium cavity, as shown in Figure 11, the same measurements were performed.

**Figure 12:**
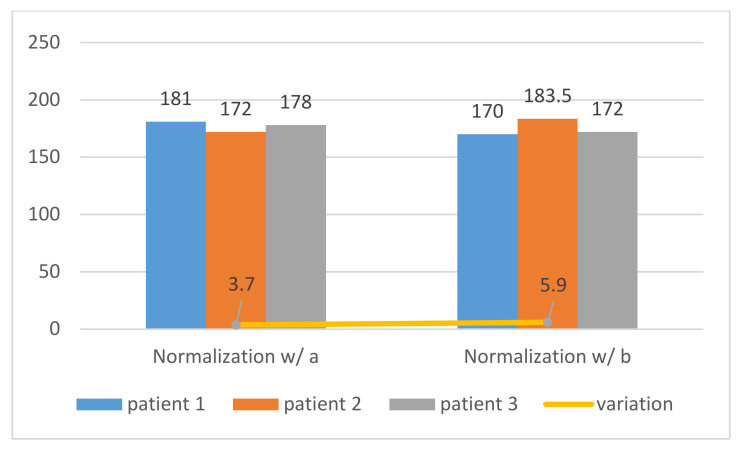
Standard deviation of binarization threshold values between normalized regions. Window width and level were kept constant.

The normalization process implemented in Figure 10 and Figure 11 was applied to three different patients, and both showed that the tests performed with the normalized ROI in the left atrium cavity (a) were more consistent and accurate than the right ventricle cavity and (b), as shown in Figure 12 and demonstrated by the standard deviation of the threshold value of region a, were lower than that of region b (3.7 < 5.9). Therefore, our image normalization process relied on a “dark” ROI to be interactively defined on the left atrium cavity.

**Figure 13:**
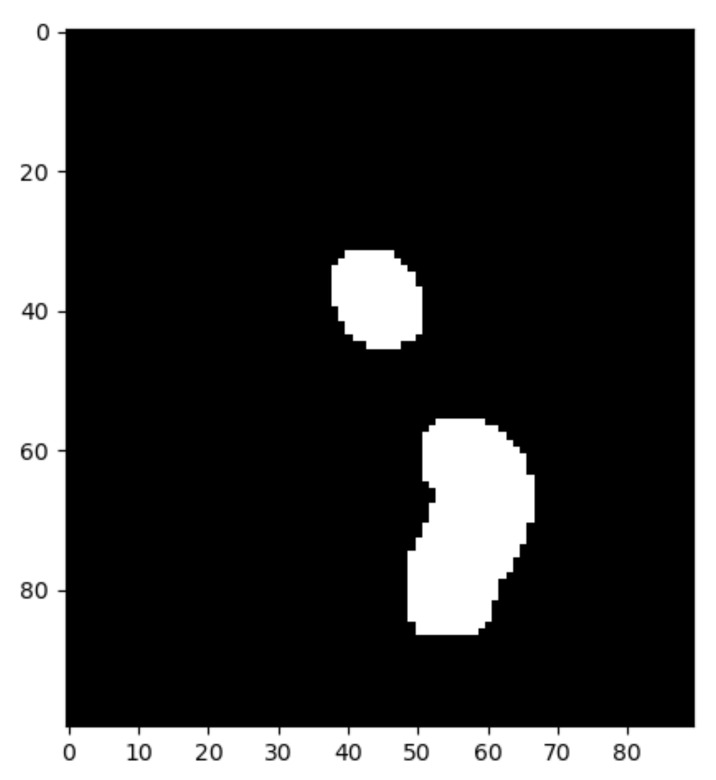
Binary image of the Aortic Valve (region of interest) where our algorithm found two areas with calcium.

After image normalization, the final binarization result with this adaptive threshold is depicted in Figure 13.

Once the calcium regions are identified (Figure 13), we proceed with defining a 2D pixel mask for each region (inspecting the ROI and keeping the 2D coordinates of the white pixels). Applying these masks to the original image, we can extract the pixel values of each sub-image that we consider as calcium, allowing us to compute some simple descriptive statistical measures, such as mean and median. Given that pixel values vary with the ultrasound properties, we subtracted the mean value of the normalization region from the values of the original image, to get normalized values regardless of the ultrasound properties. From the descriptive statistics analysis, we noticed that for all the different cases studied, the one with the lowest variation was the mean, as shown in Table 1. Taking this finding into consideration, we used this metric to validate all new cases.

These tests were performed on three different patients, where each of them performed nine echocardiographic acquisitions with different settings. These settings have interchanged between Image Compression (IC) and Ultrasound Frequency (UF), with the values of 45%, 50% and 55% for IC and 30 Hz, 50 Hz and 70 Hz for UF, resulting in 27 echocardiographic images, aiming to validate the normalization method. From these echocardiography images, 3 of them had not enough quality to be analyzed, and were discarded. In Table 1, we can see that the mean pixel values extracted from each of these three different patients are coherent, with a low absolute difference between them, showing a low standard variation as well, which suggests that our normalization method is valid.

## 3. Implementation Process and Results

**Figure 14:**
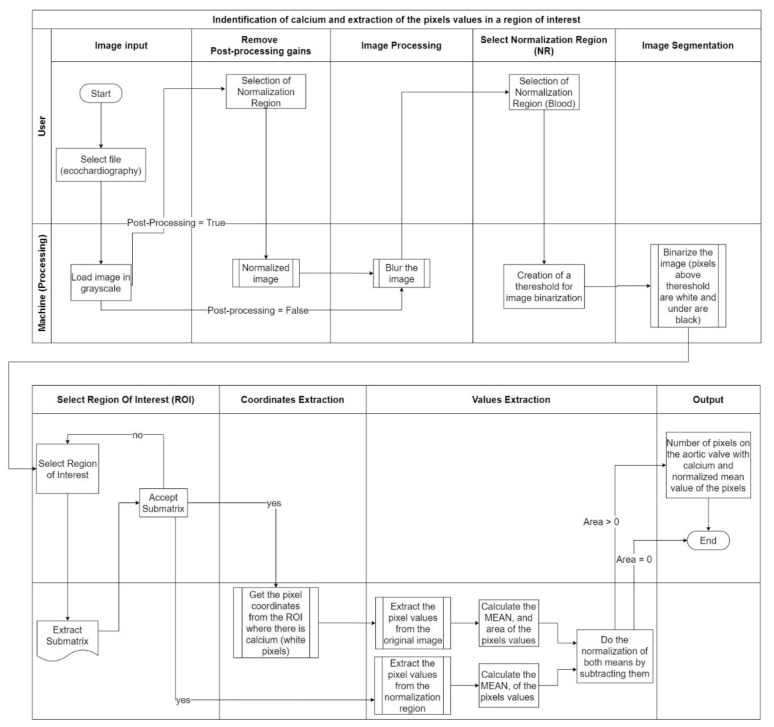
Process Description.

Considering the method developed performed in the previous section, our goal was to create an artifact that uses, as its input, the echocardiography and identifies calcium providing a score, considering the different acquisition settings. The major effort is the normalization due to the different acquisition settings. Figure 14 explains the complete process that was developed to achieve our goals.

The image processing consisted of nine different stages with two different operators—user and machine. The stages where the user is an operator, along with the image processing, will be described in this chapter.

### 3.1. Image Input

**Figure 15:**
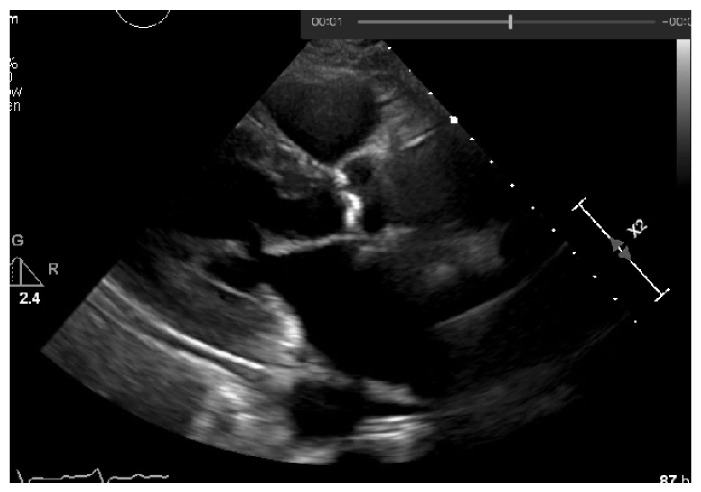
Image loaded in grayscale.

In the first stage, the user must select the image from which he intends to extract the calcium severity, allowing the machine to transform this image into grayscale and obtaining all the values scaled within the grayscale range (from 0 to 255), as shown in Figure 15.

### 3.2. Remove Post-Processing Gains

In this section, the system asks the user if the echocardiography selected has post-processing gains. If the image was subjected to such a processing stage, it is crucial to compensate them to get the real acquisition values and to ensure a more precise result. To do this, the user needs to select a region out of the sector, as represented in Figure 7.

This process will exclude the new brightness and treatment given on post-processing, achieving the original image collected by the specialist.

### 3.3. Image Processing

**Figure 16:**
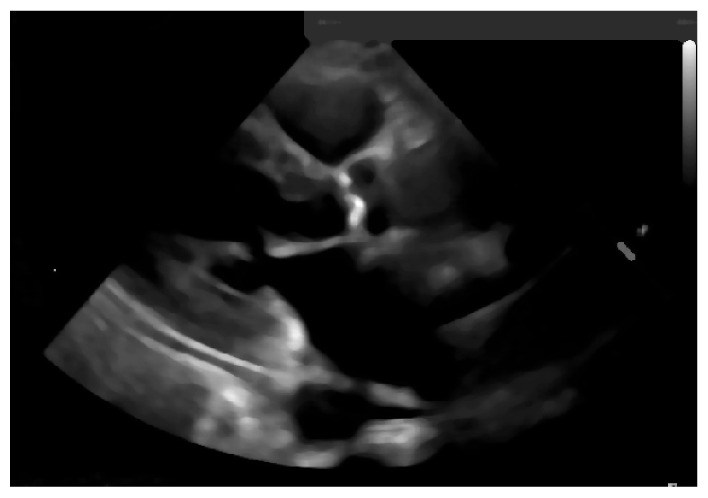
Blurred image.

Once the image is scaled, a blur will be applied to it, fading some of the image’s noise, since this process averages out rapid changes in the different pixel intensities, as shown in Figure 16.

### 3.4. Select Normalization Region and Image Segmentation

To identify the calcium, we need to compute a threshold for image segmentation. This means that the image will be binarized where the foreground (the calcium region) is white.

For the threshold, we started with a constant threshold for the pixels, of 160. Pixel values above such a figure are considered calcium and the ones under this value are non-calcium pixels (blood, fat, muscle, or fibrous).

**Figure 17:**
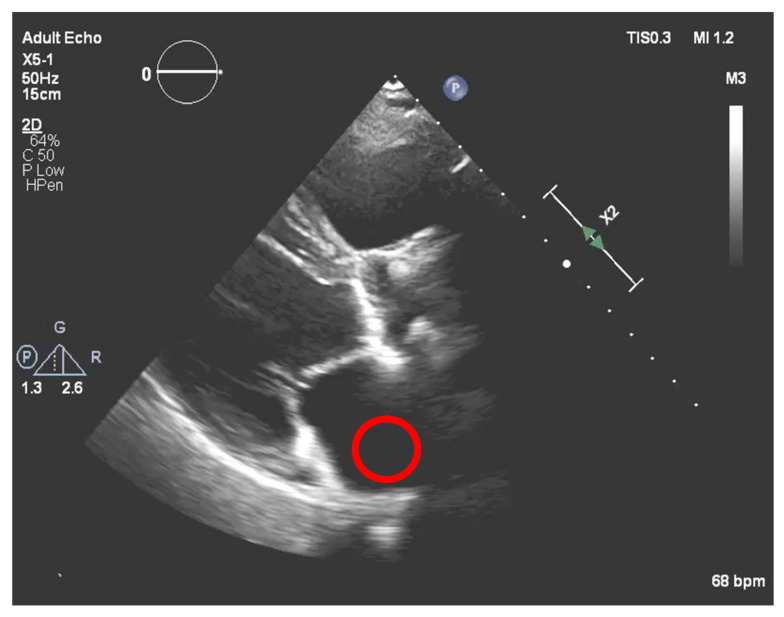
Left atrium cavity region that will create a dynamic threshold for binarization.

This initial constant threshold was weighted and defined by the experts in cardiology and echocardiography and the co-authors of this paper (AGA and LR), who have more than 20 years of experience. However, pixel intensities from echocardiographic images change with the acquisition parameter settings, such as image depth, ultrasound pulse frequency, and image compressing. Moreover, the post-processing level of gain intensity also changes the overall pixel intensity. Images collected with a combination of different parameter settings were analyzed, to test our normalization intensity values approach, and to identify an cut-offs for calcification in patients, with and without calcification, for controlling these parameters. Visual assessment by experts was used as our reference for calcium analysis. Since after collecting the echocardiography image there is a processing stage (gains are applied to the image), we would end up with an echocardiography image with different values of brightness and setting a constant binarization threshold would not provide good image segmentation. To tackle this issue, an adaptive normalization of our threshold was performed by adding the extra-brightness. This extra-brightness is taken from a region of the echocardiography that should be completely black, the left atrium cavity, as shown in Figure 17.

**Figure 18:**
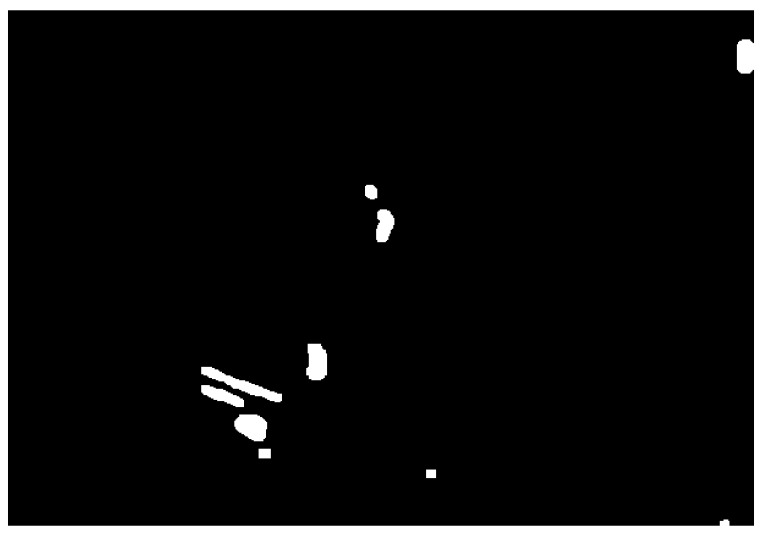
Calcium in the binarized image.

Achieving a dynamic threshold will normalize our echocardiography images, allowing our model to identify the calcium in different cases with different gains. The calcium presence can be seen in Figure 18 and is marked by a red circle.

### 3.5. Select Region of Interest

Once the image is binarized, the system asks the user to interactively select the Region Of Interest (ROI) that should contain the aortic valve. When the ROI submatrix is retrieved, we will loop over the image to extract the coordinates of the white pixels. Figure 13 depicts the end result: the submatrix with the binarized ROI.

Having the exact coordinates from where the calcium is present on the aortic valve, we will go to our original images and obtain the pixel values of the region of the aortic valve with calcium, from the coordinates extracted previously. After getting such pixel values, we will calculate the mean of our matrix of pixels. In order to get these values normalized, we compute the difference between the calcium selected from the echocardiography and the normalization region selected in Figure 17.

Regions identified as calcium—with an intensity above the dynamic threshold—will allow, after binarization, counting the number of white pixels, a proxy to the region area and an indication similar to the calcium score identified by a CT-scan. This approach requires validation performed by means of visual analysis conducted by echocardiography experts.

### 3.6. Implementation at Hospital Environment

For this study it was used Philips’s ultrasound equipment, model Epiq 7 (Eindhoven, The Netherlands).

After the performed tests, a validation set was created with 12 echocardiographic studies (from 12 different patients with calcific aortic stenosis) chosen randomly from the database, where we aimed to check our model’s accuracy in terms of classifying whether we had, or had not, a presence of calcium on the echocardiography image, based on the amount of calcification as assessed by CV-based calculation of the number of pixels, in comparison with the calcium area measured manually by planimetry in cm^2^ [24].

In Table 2, we present the results extracted from the validation set composed of these 12 samples, from which we have found a high correlation between the amount of calcium based in the number of white pixels, and the calcium area measured manually by the echocardiography experts (AGA and LR).

**Figure 19:**
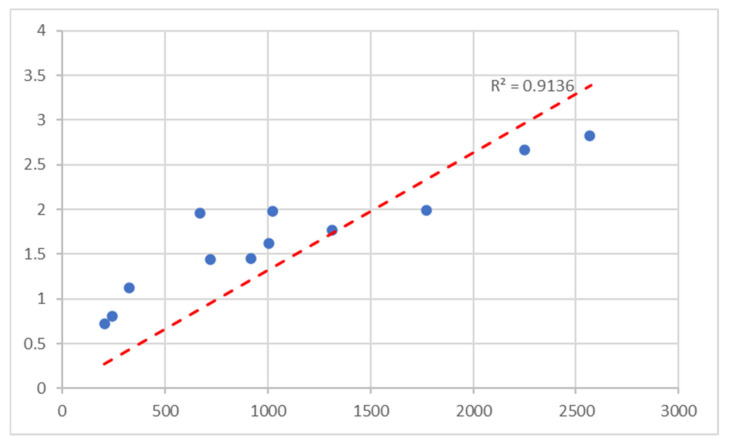
Correlation Graph, where the *Y*-axis is planimetry and the *X*-axis is the number of white pixels taken from our approach.

Pearson correlation between the number of white pixels and the area calculated by planimetry was 0.92, with a Coefficient of Determination of 0.91 and a *p*-value of 0.00048, as depicted in the correlation graph in Figure 19, showing that there is a high positive correlation [25], and a *p*-value lower than 0.001 shows that our test results are highly significant [26].

## 4. Discussion and Conclusions

We have concentrated our efforts on evaluating what the research studies are assessing regarding the score of calcium in the aortic valve and in the coronary, since from a standard coronary artery calcium computed tomography scan, we can measure the aortic valve calcification [27].

Several studies have been conducted to predict cardiovascular events, calcium being presented in the aortic valve as an accurate predictor of these events [28]. From our analysis, it became clear that all of our analyzed studies focused on utilizing CT-scans. In terms of getting coronary score calcium, this is only possible using this resource [8]. Nevertheless, this approach bears costs, namely, monetary and health costs, since it corresponds to a very invasive scan, considering that it uses radiation to extract the amount of calcium [8]. More recently, the CT-calcium score of the aortic valve has been used to identify aortic stenosis severity.

This degenerative disease evolves with ageing and is an epidemiological issue due to the high mortality, if left untreated. Literature studies performed with a cardiac CT calcium score, showed that the amount of valve calcification is related to severity and may help identify high-risk patients with an indication of valve replacement.

According to the Agatston method, calcium quantification by cardiac CT is usually presented as a calcium score and has been validated by histopathology [28]. Although it is a reference method for calcification, currently established by medical guidelines, CT is an ionizing method and its use in repeated studies should be avoided. On the other hand, echocardiography is a non-invasive, non-ionizing technique based on ultrasound that could be used for calcium detection quantification if an automated method were available and reliable. There is a lack, so far, of a reliable quantification method for calcium by using echocardiography, although this could be an appropriate method since it is free of negative effects on human health and is a widely available technique. However, the quantification of calcium based on echocardiography imaging is a challenge. Calcium is reliably detected visually by experts, but visual quantification is unreliable and is subject to variability. In this paper, calcification of the aortic valve was used in the scope of a proof-of concept study.

From the studies found, only one work regarding aortic valve calcification quantification recurring to echocardiography has been published. This means that there is a lack evaluation of the calcium score using only the echocardiography information, and only detection and prevention has been studied.

Once we add to our search CT-Scan imaging, we start obtaining published works related to quantifying the calcium, including some papers adopting deep learning. From our study we noticed that there is a larger sample in terms of studies when we are dealing with CT-scans. In fact, there are four times more papers regarding CT-scans than papers with echocardiography imaging analysis.

From our analysis, we can see that cardiac CT-scan imaging has been used for coronary calcium calcification and prognosis prediction, and some literature works adopt deep learning algorithms, while, as mentioned, there is no published work on obtaining a calcium score from echocardiography, which would avoid the disadvantages of an ionizing method such as CT-scan.

In our study, in order to avoid the use of CT-scans and the algorithm training requiring large data sets, we aimed to assess this technique for identifying and quantifying calcium in aortic valves of patients with aortic stenosis.

A Computer Vision approach enabled us to identify and quantify the amount of calcium based on echocardiography imaging analysis, in calcific aortic valve stenosis using as reference the calcium quantification by echocardiography experts.

From echocardiographic studies of calcific aortic stenosis, we analyzed the effect of changing the post-processing windowing conditions (width and level) and found a high level of agreement of intensity pixel values after normalization (by subtracting from the values of an ROI in a dark part of the image, at flow echogenicity void). An adaptive cutoff was found for pixel intensity that ensured the presence of calcium as validated by visual inspection.

Furthermore, in additional echocardiographic studies, we analyzed the pixel values when changing the settings of acquisition that affect the brightness and contrast (ultrasound frequency and compression) and the final values for pixels and normalized pixels at the reference ROI, which just showed a small difference between exams, opening the potential for wider application in the clinical setting.

Additionally, a validation set of 12 cases of calcific aortic stenosis, chosen randomly from a database, was selected for calcium quantification in the valves by assessing pixel number counting after applying the proposed cutoff for calcium. As a proxy of the amount of valve calcification, this number, in parallel to the CT calcium score, showed an excellent correlation with valve calcification measured manually via planimetry by echocardiography experts. Few studies have also addressed quantification of calcium in aortic valve and so far, only one study has shown a moderate correlation between the echocardiographic calcium amount and CT calcium score [9]. From our validation results we expect that our method will show a better performance and ability to identify the severe cases.

A limitation of this study is the small number of cases analyzed. This was in accordance, however, with its exploratory purpose. A further study should be undertaken in the future with the inclusion of a larger number of aortic valves with a large range of calcification to validate these results. Besides expert image validation, as used in this paper, a comparison with an additional validated method must be undertaken, as well as a comparison with aortic valve stenosis severity and the impact of calcification assessed by this new method on prognosis. Additionally, our method, if proved successful, will most likely be used in patients with good acoustic windows, excluding the ones with difficult ones, up to 10–12% in the clinical arena and should use a fixed number of acquisition and post-processing settings.

Moreover, this study was developed using specific echocardiographic equipment. Findings must be compared in further studies using other machines that may possibly provide different kinds of ultrasound images regarding pixel intensity and different cutoffs possibly need to be considered.

This work was a collaborative approach between a computer science university and a social science university with a medical university and a hospital to provide a solution to a real problem.

## Figures and Tables

**Table 1:** Descriptive statistics, where Echocardiography 1 to 9 belongs to patient 1, 10 to 16 belongs to patient 2, and 17 to 24 belongs to patient 3.

Echocardiography	Normalization by Mean	Normalization by Median
1	181	184
2	171	175
3	184	193
4	187	193
5	191	199
6	174	175
7	174	181
8	189	190
9	171	173
10	173	179
11	171	185
12	182	187
13	176	186
14	175	186
15	168	168
16	176	184
17	178	182
18	178	180
19	180	179
20	182	183
21	176	176
22	182	183
23	180	180
24	176	175
Standard Deviation	5.78	6.94

**Table 2:** Validation Study in a controlled patient representative—the number of white pixels (showing calcium) versus planimetry area measured manually.

	Number of White Pixels	Planimetry Area (cm^2^)	Normalized Mean
Patient 1	325	1.12	166
Patient 2	722	1.44	174
Patient 3	242	0.81	168
Patient 4	669	1.96	170
Patient 5	2251	2.67	175
Patient 6	2565	2.82	190
Patient 7	1026	1.98	188
Patient 8	917	1.45	174
Patient 9	1007	1.62	178
Patient 10	1315	1.77	172
Patient 11	206	0.72	165
Patient 12	1771	1.99	186

## Data Availability

All the data can be used on request.
